# Quality of Life in Follow-Up up to 9 Months after COVID-19 Hospitalization among the Polish Population—A Prospective Single Center Study

**DOI:** 10.3390/biomedicines12061282

**Published:** 2024-06-10

**Authors:** Ewa Pietruszka-Wałęka, Michał Rząd, Renata Rożyńska, Piotr Miklusz, Emilia Zieniuk-Lesiak, Magdalena Żabicka, Karina Jahnz-Różyk

**Affiliations:** 1 Department of Internal Medicine, Pneumonology, Allergology and Clinical Immunology, Military Institute of Medicine—National Research Institute, Szaserów 128, 04-141 Warsaw, Poland; 2 Department of Radiology, Military Institute of Medicine—National Research Institute, Szaserów 128, 04-141 Warsaw, Poland

**Keywords:** SARS-CoV-2, COVID-19, quality of life, EQ5D, 6 min walk test, long COVID, post-COVID-19 syndrome

## Abstract

The consequences of COVID-19 constitute a significant burden to healthcare systems worldwide. Conducting an HRQoL assessment is an important aspect of the evaluation of the impact of the disease. The aim of this study was to investigate the prevalence of persistent symptoms and their impact on HRQoL and health status in COVID-19 convalescents. The study group consists of 46 patients who required hospitalization due to respiratory failure and who were subsequently evaluated 3 and 9 months after hospital discharge. At the follow-up visits, the patients were asked to assess their HRQoL using the EQ-5D-5L questionnaire. The results of chest CT, 6MWT, as well as the severity of the course of COVID-19 were also considered in the analysis. The obtained results have identified fatigue as the most common persistent symptom. The majority of the convalescents reported an impairment of HRQoL in at least one domain (80% and 82% after 3 and 9 months, respectively), of which the most common was that of pain/discomfort. The presence of ongoing symptoms may affect HRQoL in particular domains. The 6MWT outcome correlates with HRQoL 3 months after hospital discharge. Therefore, it may be useful in identifying patients with reduced HRQoL, allowing early interventions aimed at its improvement.

## 1. Introduction

The COVID-19 pandemic is so far the greatest challenge in the 21st century that public health services all over the world have been confronted with. The disease is caused by the SARS-CoV-2 virus, belonging to the *Betacoronaviridae* family. It can manifest itself either as a mild respiratory infection or a severe interstitial pneumonia, as well as a multiple organ dysfunction syndrome [1]. The negative effects of a COVID-19 infection can last for many months after the disease and affect various organs. The persistence of symptoms 12 weeks after the onset of the disease, the presence of which cannot be explained otherwise, is called PACS (Post Acute COVID-19 Syndrome). The fact of experiencing a severe disease, as well as the long-term persistence of various symptoms associated with PACS, can negatively affect the well-being and quality of life of survivors [2].

Quality of life (QoL) is defined by the WHO as “individuals’ perceptions of their position in life in the context of the culture and value systems in which they live and in relation to their goals, expectations, standards and concerns” [3]. Considering the complexity of the definition above, in medical research, the term health-related quality of life [HRQoL] is usually used. Health-related quality of life covers several areas of a person’s life: the physical condition in relation to mobility, psychological state, social status, economic conditions, and somatic experiences [4].

For the assessment of the quality of life in scientific research, various self-administered questionnaires for completion by the patient themselves or with the help of their relatives are used. General questionnaires cover many aspects of life but show less sensitivity to specific disorders. This group includes, for instance, Short-Form 36 (SF-36), EQ-5D-5L (EuroQoL-5-Dimensions-5-Level), Sickness Impact Profile (SIP) or WHOQOL-BREF (World Health Organization Quality of Life Scale). A different kind of QoL assessment tool is questionnaires that focus on a specified area of study, and therefore achieve high sensitivity. Their disadvantage, however, is the lack of specific norms for the different study populations [5,6]. In the present study, we decided to use the EQ-5D-5L questionnaire mostly because of its widespread application in medical research, the possibility of measuring health-state utility, and its potential implementation for assessing both particular aspects of health (domains), as well as overall health status (VAS). Another advantage of the EQ-5D-5L questionnaire is the availability of a validated Polish language version, as well as the presence of established norms for the Polish population. An additional argument for the use of this questionnaire is its simplicity and relatively short completion time, which seems to be particularly important in the case of elderly patients, who often, due to impaired vision and emerging dementia, are not willing to read and complete long questionnaires.

The HRQoL assessment represents an important part of medical research. It helps us to investigate the impact of the disease on both the individual and on entire populations enabling us to evaluate their functional status, self-care ability, activities of daily living, and functioning in society. After all, the goal of medical care is not only to cure the patient’s disease, but most importantly, to maintain the patient’s functional status, well-being, and independence in daily functioning. The predicted quality of life often determines the patient’s decision to initiate a treatment, comply with medical recommendations, or even implement preventive habits. Moreover, measuring the quality of life in selected patient populations provides a better understanding of their problems and needs, whereas investigating the impact of a disease on the long-term quality of life may be helpful in predicting and preventing its negative effects in future patients [7,8].

Currently, when the time of the highest SARS-CoV-2 infection incidence has passed, more and more researchers around the world are focusing on investigating the long-term consequences of COVID-19 on both health status and HRQoL. Several publications regarding HRQoL of COVID-19 survivors have been recently published. Despite the different populations studied and the employment of different HRQoL assessment tools, the results of the studies are consistent in that surviving a SARS-CoV-2 infection affects negatively the quality of life of the recovered patients, both physically and, perhaps most importantly, psychologically [9,10,11,12,13,14,15,16,17,18,19,20,21,22].

To the authors’ best knowledge, to date, only a few studies regarding the quality of life of COVID-19 survivors in the Polish population have been published. In contrast to our research, previous studies were performed either with the application of HRQoL assessment scales other than the EQ-5D, or they focused on specific groups of patients.

The aim of this study was to investigate the prevalence of persistent symptoms and their impact on HRQoL, using the five-level EQ-5D scale, as well as the self-assessment of health status on the VAS scale in a group of COVID-19 convalescent patients who developed respiratory failure and required hospitalization in the acute phase of the disease. In our previous study, which examined the long-term effects of COVID-19 on respiratory function, our attention was drawn, among others, to the lack of any correlation between Pulmonary Function Test (PFTs) results and the declared HRQoL of survivors. An additional objective of the present study was to investigate the relationship between other evaluated parameters, such as the severity of COVID-19, the intensity of lesions on baseline and follow-up computed tomography (CT) of the lungs, 6 min walk test (6MWT) results, and the HRQoL. Another explored area was the correlation between the presence of specific symptoms in the acute phase of the disease (the prevalence of which we reported in a previous publication [23]) and of the persistent symptoms with the quality of life of the survivors, measured at 3 and 9 months after hospital discharge.

## 2. Materials and Methods

### 2.1. Patients and Methods

Our research is a non-interventional, prospective, observational study. The study group includes patients who were hospitalized at the Military Institute of Medicine—National Research Institute in Warsaw, suffering from respiratory failure caused by COVID-19 between October 2020 and June 2021. All the participants of the trial gave voluntary informed consent to be involved in the study. The study protocol was presented to the local Bioethics Committee, which gave its approval no. 25/WIM/2021. The inclusion and exclusion criteria are listed below in Table 1.

The study participants were followed up twice: at 3 and 9 months after hospital discharge. At each follow-up visit, the patients underwent physical examination by a physician, had a chest CT scan performed, completed the 6MWT, and were asked to fill out questionnaires regarding their current (at that time) quality of life and ongoing symptoms.

For certain analyses, the study group was divided into two subgroups according to the severity of the COVID-19 acute phase course—severe (*n* = 16) and non-severe (*n* = 30) course group. The severe course was further defined as one requiring oxygen with a flow of >15 L/min or the use of mechanical ventilation.

The HRQoL assessment was based on the EQ-5D-5L questionnaire, which comprises five dimensions of quality of life: mobility, self-care, usual activities, pain/discomfort, and anxiety/depression. Each domain of EQ-5D-5L is scored on a 5-point scale: 1—no problems; 2—slight problems; 3—moderate problems; 4—severe problems; and 5—extreme problems The second part of the assessment was based on the visual–analog scale (VAS), with the use of which patients self-assessed their health status on a scale of 0–100, with 0 indicating the worst possible state of health and 100 the best state of health imaginable [24]. In the analysis, the relevant coefficients from the parameterization model of the EQ-5D questionnaire adapted to the Polish population were used [25].

At both follow-up visits, the study participants were also requested to fill out a questionnaire regarding the presence of specific ongoing symptoms, such as the following: fatigue, fever, dyspnea, cough, muscle pain, headache, chills, smell and/or taste disorders, sore throat, diarrhea, and nausea.

Each participant of the study performed a 6MWT. The patients were instructed to walk for as long and as far as possible, on a flat, hard surface, within 6 min. The test was conducted by qualified medical professionals according to the Polish Association of Lung Diseases guidelines [26]. Vital parameters (i.e., heart rate, blood pressure, and saturation level) were assessed twice—prior to and after the completion of the test. In addition, the severity of dyspnea before and after the walk test was rated from 0 to 10 according to Borg’s scale [26]. The test outcomes were measured in meters, then they were converted and presented as % of the predicted value (estimated by the formula proposed by Gibbons et al.) [27].

Baseline and follow-up chest CT scans were evaluated by an experienced radiologist using the CT COVID severity score (CT CSS). Points were assigned from 0 to 25 according to the severity of the interstitial lesions (from 0 to 5 points for each lobe) [28].

### 2.2. Statistical Analysis

The analysis of the data was performed using Statistica (version 13, TIBCO, Palo Alto, CA, USA), R software (version 4.2.3, The R Project for Statistical Computing, Aucland, New Zeland), and MS Excel (version 2311, Microsoft Corporation, Redmond, WA, USA). Quantitative variables were presented as means and 95% confidence intervals (95% CI) or medians and quartile ranges (IQR) for data with normal or non-normal distribution, respectively. Nominal data were presented as numbers and percentages. The Shapiro–Wilk test was used to verify normal distribution. The groups were compared in relation to ordinal or quantitative data with a non-normal distribution using the Mann–Whitney U test. For the comparison of the distribution of nominal variables between groups, the chi-square test was used, and (where appropriate) additionally the Yates correction or Fisher’s exact test were used. For dependent data, the Student’s *t*-test or the Wilcoxon signed-rank test were used for parametric and non-parametric data, respectively. The correlation of quantitative data with non-normal distribution was calculated using the rho-Spearman correlation. Quality of life coefficients were determined based on data on the outcomes in each domain of the EQ-5D questionnaire and with the use of the corresponding coefficients from the parameterization model of the EQ-5D questionnaire developed for the Polish population [25]. A two-sided *p*-value below 0.05 was considered statistically significant.

## 3. Results

The study group consisted of 46 patients (48% female, 52% male), for whom the median age was 63 years (IQR 53–69). The median length of hospital stay was 20 days (IQR 11–31 days). Radiological severity as measured by the CCTS scale for the acute phase, follow-up at 3 and 9 months were 15 (IQR 12–17), 8.5 (IQR 5–11), and 6 (IQR 4–9), respectively. For the purpose of some evaluations, the patients were further categorized into severe and non-severe subgroups (definition above). The median age of the severe course group was 64 years (IQR 57–66), while that of the non-severe group was 63 years (IQR 57–72). The median length of hospitalization was 32 days (IQR 23–48 days) and 12 days (IQR 10–20 days) for the two groups, respectively (Table 2).

### 3.1. Persistent Symptoms

**Figure 1:**
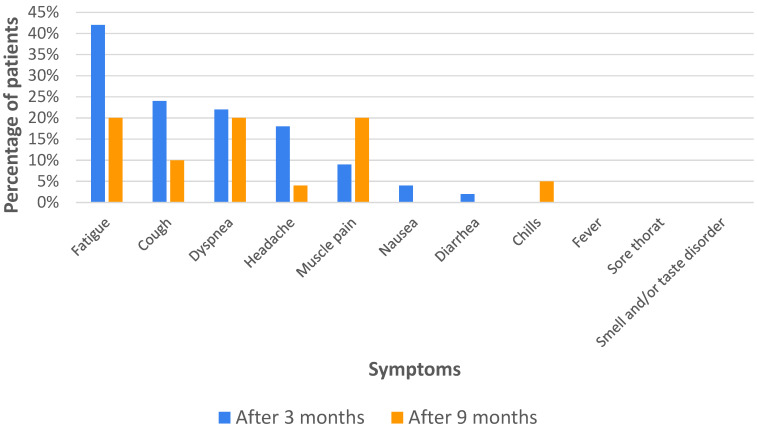
Symptoms persisting at 3 and 9 months after hospital discharge.

At the first follow-up visit, 3 months after hospital discharge, almost 58% of the patients suffered from at least one ongoing symptom, of which 22% reported having three or more symptoms at the same time. The most frequently reported persistent symptom was fatigue (42% of the patients), followed by cough (24%), shortness of breath (22%), and headache (18%). Far less commonly, the patients reported muscle pain (9%), nausea (4%), diarrhea (2%), as well as smell and/or taste distortion (2%). None of the surveyed patients reported any occurrence of fever, chills, or sore throat at that time (Figure 1).

At the second follow-up visit, 9 months after hospital discharge, only 35% of the patients reported persistent symptoms, with only 15% reporting simultaneous occurrence of three or more symptoms. Among the most frequently reported symptoms were, as before, fatigue, followed by dyspnea and myalgia (each 20%); chronic cough persisted in 10% of the patients, while headache and chills occurred in 5% of the respondents, respectively (Figure 1).

The results of the study showed no significant differences in the incidence of persistent symptoms at 3 and 9 months after hospital discharge between the severe and non-severe subgroups.

### 3.2. Health-Related Quality of Life

All of the patients, at both follow-up visits, were asked to complete an HRQoL questionnaire. At the first follow-up, 3 months after hospital discharge, as much as 80% of the respondents reported impaired quality of life (at any level) in at least one of the 5 domains, 44% of the patients reported impaired quality of life in three or more of the domains, and 4% of the respondents declared at least slight problems in all of the domains. The average quality of life using the VAS scale was assessed at 73% (95% CI: 69–78%).

Most patients reported impairment in the HRQoL in the domain of pain/discomfort—as much as 66%, of which 36% rated the degree of impairment in this domain as mild, 25% as moderate, and only 4.5% as severe. Far fewer respondents declared any level of impairment of HRQoL in the domains of anxiety/depression (50%), mobility (49%), and usual activities (40%). Only 14% of the patients indicated deterioration in the domain of self-care (of which 7% rated its degree as mild and 7% as moderate).

At the second follow-up visit, 9 months after hospital discharge, deterioration in the quality of life in at least one of the domains was reported by 82% of the respondents. Interestingly, this is 2% more than at the first follow-up. Nearly 12% of the respondents declared impairment of the quality of life in only one domain, while the percentage of patients reporting impairment in two, three, or four domains was 24, 29, and 18%, respectively. The average rating of the patients’ own general health status using the VAS scale was slightly higher than 3 months after hospital discharge, with a score of 76% (95% CI: 68–85%); however, this difference was not significant.

**Figure 2:**
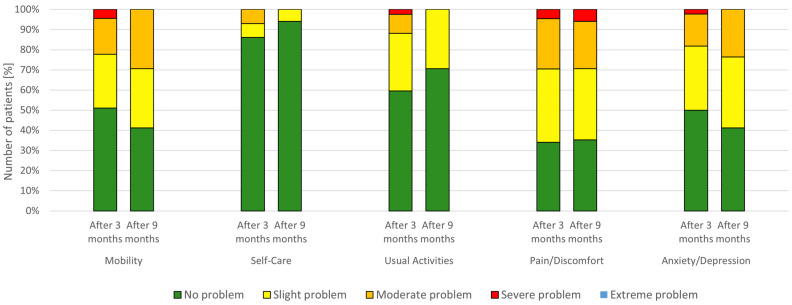
HRQoL at 3 and 9 months after hospital discharge.

Similarly to the results obtained after 3 months, the most frequently affected domain was that of pain/discomfort (65% of the patients), followed by the domains of mobility and anxiety/depression (both 59%). The domain of usual activities was impaired in 29% of the patients. As previously, the least affected domain was self-care (only 6% of the recovered patients)—Table 3, Figure 2. No significant differences in the level of HRQoL impairment in specific domains were observed between the 3-month and 9-month follow-ups.

### 3.3. Occurrence of Specific Symptoms and HRQoL

#### 3.3.1. Occurrence of Different Symptoms during the Acute Phase of COVID-19 and HRQoL

At both follow-ups, neither the number of different types of symptoms present in the acute phase of the disease nor the occurrence of a specific symptom in the acute phase of the disease had any significant impact on the quality of life in any of the assessed domains.

#### 3.3.2. Occurrence of Selected Persistent Symptoms and HRQoL after 3 Months

The results of the study revealed a significant negative impact of the occurrence of symptoms persisting 3 months after hospital discharge on specific domains of quality of life:

- The presence of dyspnea shows a negative correlation with HRQoL in the mobility (*p* < 0.01) and anxiety/depression domains (*p* <0.05);
- The presence of muscular pain correlates with deteriorated HRQoL in the domains of mobility (*p* < 0.05), anxiety/depression (*p* = 0.01), and pain/discomfort (*p* < 0.05);
- The occurrence of fatigue is associated with worsening of HRQoL in the domains of mobility (*p* < 0.01), anxiety/depression (*p* < 0.001), pain/discomfort (*p* < 0.05), usual activities (*p* < 0.05), and general health status on the VAS scale (*p* < 0.05).

#### 3.3.3. Occurrence of Selected Persistent Symptoms and HRQoL after 9 Months

Investigation of the impact of the occurrence of particular symptoms persisting at the second follow-up visit (9 months after hospital discharge) on the quality of life declared by respondents at that time showed a significant negative correlation between the following:

- The presence of dyspnea had a negative correlation with the VAS total health score (*p* < 0.05);
- The occurrence of fatigue was associated with a deteriorated quality of life in the domains of usual activities (*p* < 0.05) and pain/discomfort (*p* < 0.005);
- Muscle pain was inversely related to HRQoL in the domain of self-care (*p* < 0.05) and pain/discomfort (*p* < 0.05);
- The presence of cough negatively correlated with HRQoL in the domains of usual activities (*p* < 0.05) and pain/discomfort (*p* < 0.05).

### 3.4. Severity of COVID-19 and HRQoL

There was no significant correlation between the severity of the course of the disease and the quality of life in any of the assessed checkpoints.

### 3.5. Radiological Findings and HRQoL

The results of this study revealed no significant relationship between the CT CSS radiological lesion intensity score (at any of the assessed moments) and the overall quality of life score at 3 or 9 months after hospital discharge.

### 3.6. Sex-Related Differences in HRQoL

At follow-up 3 months after hospital discharge, women reported significantly greater deterioration of the quality of life in the domains of mobility and anxiety/depression than men (*p* < 0.05). No such relationship was observed at follow-up 9 months after hospital discharge.

### 3.7. 6MWT Results and HRQoL

The study findings revealed a positive correlation between the 6 min walk test outcome (% of predicted value) and the total HRQoL index assessed at the first follow-up 3 months after hospital discharge (*p* < 0.001, r = 0.51). No correlation between these parameters assessed 9 months after hospital discharge was found.

Furthermore, it was found that a better outcome in the 6MWT (% of predicted value) obtained 3 months after hospital discharge was associated with better quality of life in the domains of mobility (*p* < 0.001, r = 0.57) and self-care (*p* < 0.05, r = 0.35), as well as a higher VAS score (*p* < 0.05, r = 0.34) at the first follow-up visit.

Similarly, 9 months after hospital discharge, a higher 6MWT outcome (% of predicted) correlated with a better quality of life in the domain of mobility (*p* < 0.001, r = 0.71) and a higher VAS score (*p* < 0.005, r = 0.64).

In addition, at the 9-month follow-up, the severity of dyspnea following the 6MWT, as assessed by the Borg scale, showed a negative correlation with the VAS score (*p* < 0.05, r = −0.53). No such correlation was found in the 3-month follow-up (Table 4).

Interestingly, women achieved significantly worse results (% of pred.) in the 6MWT than men (*p* < 0.001). Mean (SD), respectively: 60.1% (13.9%) vs. 78.0% (9.1%).

## 4. Discussion

The COVID-19 pandemic has left its mark on the health and mentality of millions of people around the world. The high contagiousness of the virus and significant mortality rate widely commented on on social media, as well as forced isolation, restrictions in access to medical care, concern about the future, and finally, personal and family tragedies had a negative impact on people’s HRQoL.

Now that widespread vaccination, natural immunization processes, as well as improved detection, isolation, and treatment methods have managed to control the rapid spread of infection, it is time to confront the long-term consequences of the pandemic. While the physical aspects of COVID-19 complications have been widely studied and discussed, far less attention has been paid to date to the aspect of the persistent deterioration in the HRQoL of the recovered.

The purpose of our research was to investigate the impact of COVID-19 infection and related factors on the quality of life of the convalescents in the Polish population measured at 3 and 9 months after hospital discharge. An additional aim of the study was to explore possible factors affecting the degree of deterioration in the quality of life and to search for correlations between selected long-term symptoms, as well as the results of various additional examinations, with quality of life. Thereby, this might help to identify patients whose overall quality of life, as well as its particular domains, may have deteriorated significantly, and enable us to take preventive actions during the acute infection, as well as to implement therapeutic interventions at later stages.

To the best of the authors’ knowledge, so far only one study similar to ours has been conducted in the Polish population which was a telephone survey conducted by Koźlik et al. based on the assessment of HRQoL via SF-36 questionnaire in COVID-19 convalescents from the Silesia region [29]. All of the other Polish studies focused on specific, narrow groups of subjects—such as women [30], kidney transplant patients [19], or the elderly [31]. Reported results vary between studies, and also differ in many aspects from the findings that we obtained, as discussed further below.

The results of our study have shown that at least one persistent symptom was present in 58% of the convalescents 3 months after hospital discharge and in 35% 9 months after hospital discharge. This indicates the persistent nature of the disorder and the occurrence of long-term, burdensome, and difficult-to-treat chronic symptoms. Meanwhile, the reduction in the percentage of individuals declaring persistent symptoms during follow-up indicates a gradual, slow withdrawal of those.

The majority of previously published studies have reported a higher incidence of persistent symptoms in various time intervals [10,17,19,20,22]. The highest percentage of patients, i.e., 74%, that declared the presence of ongoing symptoms, 90 days after the onset of the disease, was reported in a British study by Arnold et al. [22].

On the other hand, an Italian research by Righi et al. reported much lower rates with at least one persistent symptom being present in 31% of the patients 3 months after symptom onset and in 20% after 9 months [21]. Such differences in the obtained results may be due to medical reasons (such as disease severity, treatment administered, etc.), but they may also reflect differences in mentality, attitude towards life, financial status, and access to medical service between the study populations.

The results we obtained revealed that the most common ongoing symptom was fatigue, declared by 42% of the convalescents at the follow-up after 3 months and 20% after 9 months. The fact that fatigue was the most frequently reported persistent symptom is not unexpected, as it is a common complaint after an infection involving the respiratory system. In addition, a number of factors associated with a SARS-CoV-2 infection can indirectly cause fatigue, such as neurological complications and prolonged hospitalization resulting in impaired muscle strength, as well as psychological aspects such as anxiety, sleep disorders, and PTSD, which in the long term may result in physical exhaustion, interpreted by the patients as fatigue. Our result is consistent with previously published studies, which also reported fatigue as the most common persistent symptom [10,11,17,19,21,22]. Interestingly, despite the study group consisting of relatively severely ill patients, the incidence of fatigue was lower than in some of the previously released publications [10,11]. As mentioned before, these differences likely reflect the varying severity of the course of the disease, differences in the applied treatment, or specific characteristics of different patient populations. Contrary to other publications, the second most common persistent symptom was cough (24% of the respondents after 3 months, but only 10% after 9 months). In third place came dyspnea (22% after 3 months and 20% after 9 months), which was reported by both Jacobs et al. and Garrigues et al. as the second most common symptom [10,11]. These results indicate that in our study group, cough was mainly present in the initial phase of convalescence, while dyspnea persisted for a long time and at the 9-month follow-up was still reported by 1/5 of the respondents. Perhaps, this could be also explained by the fact that in our study group, there were many patients with severe COVID-19 who required mechanical ventilation, HFNOT with high oxygen flow, and extended oxygen therapy. Prolonged cough may have been not only a symptom of the primary infection in these patients but also a complication associated with the treatment. The high prevalence of cough in the early recovery period is also observable in the results of an Italian study by Belli et al., which showed the presence of cough in 90% of the patients at the time of hospital discharge [12].

The results of our study indicate that 3 months after hospital discharge, as many as 80% of the recovered patients had impaired quality of life in at least one domain, while 9 months after discharge this percentage rose to 82%. These results make us realize how many among the COVID-19 survivors require special attention and perhaps targeted interventions to improve their HRQoL. An interesting phenomenon of an increasing percentage of patients reporting poor quality of life between two checkpoints was observed both in our study, and the one performed by Gaspar et al. in which at 3 months after discharge, 65.8% of the respondents declared that their quality of life was impaired, at 6 months—69.2%, while at 9 months this percentage decreased to 55.4% [17]. This emphasizes the long-term nature of post-COVID disorders and the long time required for their resolution. A possible explanation for this phenomenon can be found in the pandemic-related restrictions that negatively affected the mental sphere (and therefore the overall HRQoL level), as well as in the difficulties in access to medical care and rehabilitation or the limited contact with relatives.

In general, the percentage of patients declaring impaired HRQoL after COVID-19 varies significantly between publications, ranging from 31% at 3 months after the disease, up to 100% among patients who have experienced ARDS [9,13,16].

Therefore, considering the significant differences in achieved results, it is evident that this issue requires an in-depth analysis performed on a large group of patients.

The most frequently affected domain of HRQoL according to the results that we obtained was pain/discomfort, which is in line with the results of other studies where the EQ-5D-5L scale was used [19,32]. However, some studies have also identified the domains of mobility [14] or anxiety/depression [17] as the most commonly affected, while in our study anxiety/depression was the second most commonly impaired domain. The Chinese study by Huang L et al. found that post-COVID patients experienced more problems in the domains of mobility, pain/discomfort, and anxiety/depression than healthy controls [20]. The fact that pain/discomfort and anxiety/depression were among the most frequently impaired domains of HRQoL could suggest that the disease had a greater impact on the psychological rather than the physical aspect of HRQoL. This hypothesis may be potentially strengthened by the results obtained in an Austrian study utilizing the SF-36 questionnaire, in which quality of life impairment in the physical sphere was reported by 7% of respondents, while in the mental sphere by as many as 18%, and in both spheres by 7% [13].

Also, similar to the results of previously published research, the least affected domain in our survey was self-care [9,17,32]. This may lead to the conclusion that although the HRQoL of the survivors was impaired in many aspects, it did not affect significantly their ability to take care of themselves. Such results might indicate that, rather than impairment in physical functioning, the main reason for the deterioration of the HRQoL of the recovered is the disease-related isolation, loneliness, and inability to perform usual daily activities due to pandemic-related restrictions, which all lead to the development of anxiety/depressive disorders.

The VAS score that we obtained in our study group, i.e., 73% and 76% (after 3 and 9 months, respectively), seems to be in accord with the results of other studies in which, depending on the time interval and the population studied, the rate was between 64.83% and 80% [11,14,16,19]. An increase of a few percent in VAS scores between the two checkpoints may reflect a slow, gradual recovery process. This also supports the hypothesis that the decrease in quality of life is more likely related to the psychological sphere than the physical, as, despite the increase in VAS, the HRQoL rating decreases during follow-up.

Similarly to some previous publications, our results do not show any significant association between the severity of the course of COVID-19 and the HRQoL of the recovered patients [11,13,15,17]. However, there are also studies that have proven the opposite [22,31]. It should be also noted that our study group does not include patients with a very mild or asymptomatic course, as only patients with respiratory failure were hospitalized during the time from which the data originates, due to a health-care system overload, and therefore the lack of the above-mentioned relationship may be a result of the homogeneity of the study group. Another interesting finding is the fact that at the 3-month follow-up, women reported a worse HRQoL in the mobility and anxiety/depression domains in comparison to men. It seems that women are more vulnerable to stress and associated mental disorders, although it is also possible that men, due to social factors, were less likely to admit in the survey that they experienced troubles in the mental sphere. Problems in the mobility domain in women may be related to their genetically reduced muscular mass which may slow down the rehabilitation process. Another potential explanation for this phenomenon may be related to the aforementioned higher prevalence of anxiety disorders and depression in women which results in reduced physical activity and lower motivation for rehabilitation. A number of previously published studies, including those conducted among the Polish population, have also reported greater deterioration in HRQoL [15,29,30], a lower VAS score [14], and a higher prevalence of persistent symptoms among women [10].

Several authors have already emphasized the negative relationship between the presence of persistent PACS symptoms and health-related quality of life [10,15,21,30]. Considering that many studies have not found any relationship between disease severity and HRQoL, it can be concluded that a long-term presence of symptoms, even the relatively mild ones, has a stronger negative impact on patients’ HRQoL than the intensity of symptoms, i.e., a severe, though relatively shorter, acute phase of the disease.

In our study, we explored the connections between the presence of persistent symptoms and the overall HRQoL score, as well as its particular domains. According to the results that we obtained from the follow-up at 3 months, persistent dyspnea may be indicative of prolonged poor exercise tolerance, which results in reduced quality of life in the domain of mobility. What is more, dyspnea, also called “shortness of breath”, being a very unpleasant sensation may lead to the escalation of stress or panic and, as a consequence, to lower HRQoL in the domain of anxiety/depression.

Similarly, muscle pain, probably associated with muscle atrophy and weakening after a serious illness, correlates with lower quality of life in the domain of mobility and, understandably, in the domain of pain/discomfort. Reduced mobility and persistent pain, on the other hand, can lead to psychological disorders, which is reflected in an impaired HRQoL in the domain of anxiety/depression.

The symptom affecting the greatest number of HRQoL domains is the subjective feeling of fatigue, which apart from the mobility, pain/discomfort, and anxiety/depression domains affects negatively also the general health status assessment on the VAS scale. This phenomenon can be explained in two ways. It is possible that the patients with more comorbidities who score lower on the VAS scale are more likely (for various reasons) to experience fatigue. On the other hand, persistent fatigue lasting several months, which patients have not experienced before, may significantly affect their assessment of their own health.

The outcomes of our study at 9 months after discharge from the hospital show that the presence of dyspnea correlated with a lower VAS health score. This correlation was not evident in the results obtained at 3 months, which may suggest that only when dyspnea persists for an extended period does it significantly worsen the quality of life.

Another association we found was between the presence of fatigue 9 months after discharge and a decrease in HRQoL in the domain of usual activities. The same correlation was observed at the first follow-up, which may reflect the fact that the patients experiencing fatigue, due to a lack of energy, find it difficult to perform daily activities. In addition, persistent fatigue may have moderated pain perception, leading to a correlation of this symptom with worsening quality of life in the pain/discomfort domain.

Persistent muscle pain, similarly to the findings at the 3-month follow-up, correlated with lower HRQoL in the domain of pain/discomfort, which further interfered with the patients’ daily functioning and negatively affected their ability of self-care which may have led to their dependence on a third party.

Contrary to the results of the first follow-up, at 9 months, the presence of persistent cough corresponded with lower HRQoL in the domain of usual activities, indicating that prolonged, intractable cough negatively influenced the daily functioning of the recovered patients. Interestingly, persistent cough showed also a correlation with lower HRQoL in the domain of pain/discomfort. The reason for this association could be intercostal muscle pain caused by repeated severe coughing fits.

Neither the severity of the lesions in the follow-up chest CTs, nor the results of the pulmonary function tests [23] showed any significant correlation with the quality of life of the convalescent patients. However, at the follow-up after 3 months, we did find such correlation for the outcome of the 6 min walk test (% of predicted value). This is a very interesting and remarkable correlation, as the 6MWT is a cheap, easily accessible, and uncomplicated diagnostic tool.

In addition, the 6MWT outcome also shows a positive correlation with HRQoL in the domain of mobility and the overall VAS score, as well as with a better score in the self-care domain at 3 months, which, although least impaired, appears to be crucial to the patient’s independent existence.

The correlation between the 6MWT outcome and HRQoL in the mobility domain seems to be a natural consequence of patients’ better physical fitness—both baseline and achieved through a faster process of recovery or intensive rehabilitation. It is possible that, as a consequence of better general fitness, patients experienced less problems in their everyday routines, which was reflected by better ratings in the domain of self-care activities and, as a further consequence, a better overall health assessment on the VAS scale.

The negative correlation between the degree of dyspnea after 6MWT and the VAS score at the 9-month follow-up, as well as its absence at the 3-month follow-up, may suggest that only long-term persistent symptoms negatively affect the patients’ assessment of their own health. This finding is coherent with the aforementioned relationship between persistent dyspnea and the worsening of VAS scores after 9 months.

Moreover, the results demonstrated that women performed significantly worse in the 6MWT (according to the comparison of % of predicted values, adjusted for sex). This finding is in line with the previously mentioned impairment of HRQoL in the mobility domain among women, and also supports the hypothesis that the 6MWT outcome corresponds with HRQoL rating in the mobility domain.

Like any other instrument, the 6MWT has certain limitations, e.g., it is not suitable for people with significant musculoskeletal disabilities or severe neurological disorders. Nevertheless, due to its simplicity, it can be a useful way to assess patients’ physical fitness, while its correlation with the HRQoL level enables us to identify the patients with significantly impaired HRQoL who require intervention for its improvement. Moreover, the advantage of the 6MWT is the immediate result, which permits quick decision making and the implementation of interventions, without unnecessary delay, which can be particularly important in a situation of health-care system overload.

## 5. Study Limitations

Like any other study, this one also has several limitations. First of all, the relatively small study group may not reflect the diversity of the entire population, so extrapolated conclusions are at a significant risk of bias. Secondly, Poland is an economically heterogeneous country and access to medical care varies between regions. As most of the patients hospitalized at Warsaw Military Institute originated from the Warsaw metropolitan area, the obtained results may not be representative of the entire nation. Therefore, it would be recommended to conduct a multi-center study involving a larger group of patients from various regions.

Thirdly, due to the overload of the medical-care system during the pandemic, only patients with respiratory failure were admitted to the hospital, which means that the study group does not include patients with asymptomatic and mild course of the disease. Moreover, the period of patients’ involvement in the study covers only the first wave of the pandemic in Poland, hence the obtained results are not generalizable to all COVID-19 cases, and especially not to those following vaccination.

Another possible contributing factor could be the motivation of the patients who agreed to participate in the study and attend follow-up visits. This may have been a group of patients who wanted to improve their poor quality of life or deal with complications of the disease, persistent symptoms, etc. This may have influenced the selection of the study group, and hence, the obtained results.

Another important limitation of our study is the absence of a control group. This makes it impossible to directly relate its results to a population uninfected with SARS-CoV-2 living under the same conditions and at the same time. What is more, this makes it difficult to identify independent factors that may have negatively affected the entire population at that particular time, such as the necessity to rearrange daily life, limited social interactions, fear of getting infected, etc.

These limitations, however, are diminished by the 9-month prospective follow-up, a comprehensive assessment of the patient’s health status based on subjective symptoms, functional and radiological results, and an evaluation of the subsequent clinical presentation, as well as their relation to the patient’s HRQoL.

## 6. Conclusions

Although this study has some limitations, mostly due to the small size of the study group and the non-inclusion of asymptomatic patients, the results presented here provide background for a number of noteworthy conclusions. Most importantly, the results of our research demonstrate the existence of a significant group of COVID-19 convalescents who suffer from bothersome symptoms that persist for many months after the end of the acute phase of the disease. The most common of them is fatigue. What is more, the COVID-19 survivorship and limitations associated with the pandemic may negatively affect the health-related quality of life of the recovered patients in many aspects. Pain/discomfort is the most frequently impaired domain of HRQoL, while self-care remains the least commonly affected. Moreover, the female gender may be considered a risk factor for a lower quality of life in the mobility and anxiety/depression domains in the early phase of the recovery. These findings are of great importance, as it is likely that this group of patients will require special attention and further interventions to improve their HRQoL.

An additional, interesting finding of the study is the positive correlation between the outcome of the 6MWT and the HRQoL. The presence of this relationship may allow clinicians to use the 6MWT in their daily practice as a simple, non-invasive screening tool for the detection of the deterioration of HRQoL among COVID-19 convalescents.

To summarize, the HRQoL is an important aspect in the evaluation of the health status of COVID-19 convalescents, and it is advisable to proactively search for areas in which it may have deteriorated in order to conceptualize interventions for its improvement. Special attention should be given to individuals in risk groups and those experiencing persistent symptoms, the presence of which may correlate with an impaired HRQoL.

## Figures and Tables

**Table 1:** Inclusion and exclusion criteria for participation in the study.

Inclusion Criteria	Exclusion Criteria
- SARS-CoV-2 virus infection confirmed by PCR test - Respiratory failure - Hospitalization at the Military Medical Institute—National Research Institute in Warsaw between October 2020 and June 2021 - Adults ≥18 years of age - Polish nationality - Acknowledging the information for study participants and giving informed consent to participate in the study	- Children <18 years of age - Incapacitation, imprisonment, active service soldiers - Diagnosis of an active malignancy - Severe mental illness that could make the ability to give informed consent questionable - Inability to attend follow-up appointments - Refusal to give informed consent - Death during baseline hospitalization

**Table 2:** Characteristics of the study group by subgroups.

	Total	Severe	Non-Severe	Significance
Number (*n*)	46	16	30	-
Median age (years)	63 (IQR 53–69)	64 (IQR 57–66)	63 (IQR 57–72)	*p* > 0.05
Median length of hospital stay (days)	20 (IQR 11–31)	32 (IQR 23–48)	12 (IQR 10–20)	*p* < 0.05
Sex (female, male)	F-48%, M-52%	F-62%, M-37%	F-40%, M-60%	*p* > 0.05

**Table 3:** HRQoL at 3 and 9 months after hospital discharge—results for each domain of the EQ-5D-5L questionnaire.

HRQoL Domain	Follow-Up	No Problem	Slight Problem	Moderate Problem	Severe Problem	Unable to Do
Mobility	After 3 months	51.11%	26.67%	17.78%	4.44%	0.00%
	After 9 months	41.18%	29.41%	29.41%	0.00%	0.00%
Self-Care	After 3 months	86.36%	6.98%	6.98%	0.00%	0.00%
	After 9 months	94.12%	5.88%	0.00%	0.00%	0.00%
Usual Activities	After 3 months	59.52%	28.57%	9.52%	2.38%	0.00%
	After 9 months	70.59%	29.41%	0.00%	0.00%	0.00%
Pain/Discomfort	After 3 months	34.09%	36.36%	25.00%	4.55%	0.00%
	After 9 months	35.29%	35.29%	23.53%	5.88%	0.00%
Anxiety/Depression	After 3 months	50.00%	31.82%	15.91%	2.27%	0.00%
	After 9 months	41.18%	35.29%	23.53%	0.00%	0.00%

**Table 4:** Relationship between 6MWT outcome, the severity of dyspnea during the test, and HRQoL.

Variable	Mobility		Self-Care		Usual Activities		Pain/ Discomfort		Anxiety/ Depression		VAS	
	r	*p*	r	*p*	r	*p*	r	*p*	r	*p*	r	*p*
Follow-up after 3 months												
6MWT (% of pred.)	−0.57	<0.001	−0.35	0.026	−0.11	0.521	−0.25	0.109	−0.29	0.073	0.34	0.029
Dyspnea pre-6MWT (Borg’s scale)	0.09	0.583	−0.09	0.560	0.15	0.378	0.02	0.900	0.22	0.177	0.16	0.310
Dyspnea post-6MWT (Borg’s scale)	−0.05	0.765	−0.18	0.280	0.24	0.155	0.18	0.247	0.22	0.170	−0.01	0.935
Follow-up after 9 months												
6MWT (% of pred.)	−0.71	0.001	−0.36	0.159	−0.11	0.687	0.14	0.604	−0.01	0.984	0.64	0.005
Dyspnea pre-6MWT (Borg’s scale)	0.24	0.360	0.38	0.135	0.32	0.212	0.60	0.011	0.34	0.178	−0.19	0.475
Dyspnea post-6MWT (Borg’s scale)	0.55	0.023	0.39	0.118	0.42	0.093	0.45	0.070	0.18	0.496	−0.53	0.030

## Data Availability

The data are available in the repositories of the Military Institute of Medicine in Warsaw and can be made available for legitimate interest after contacting the corresponding author.
